# Monosodium Glutamate Reduces ^68^Ga-PSMA-11 Uptake in Salivary Glands and Kidneys in a Preclinical Prostate Cancer Model

**DOI:** 10.2967/jnumed.118.215350

**Published:** 2018-12

**Authors:** Etienne Rousseau, Joseph Lau, Hsiou-Ting Kuo, Zhengxing Zhang, Helen Merkens, Navjit Hundal-Jabal, Nadine Colpo, Kuo-Shyan Lin, François Bénard

**Affiliations:** 1 Department of Molecular Oncology, BC Cancer Research Centre, Vancouver, British Columbia, Canada; and; 2 Department of Radiology, University of British Columbia, Vancouver, British Columbia, Canada

**Keywords:** prostate-specific membrane antigen, ^68^Ga-PSMA-11, monosodium glutamate, salivary glands, kidney

## Abstract

We evaluated the ability of monosodium glutamate (MSG) to reduce salivary and kidney uptake of a prostate-specific membrane antigen (PSMA) radioligand without affecting tumor uptake. **Methods:** LNCaP tumor–bearing mice were intraperitoneally injected with MSG (657, 329, or 164 mg/kg) or phosphate-buffered saline (PBS). Fifteen minutes later, the mice were intravenously administered ^68^Ga-PSMA-11. PET/CT imaging and biodistribution studies were performed 1 h after administration. **Results:** Tumor uptake (percentage injected dose per gram [%ID]) was not statistically different between groups, at 8.42 ± 1.40 %ID in the 657 mg/kg group, 7.19 ± 0.86 %ID in the 329 mg/kg group, 8.20 ± 2.44 %ID in the 164 mg/kg group, and 8.67 ± 1.97 %ID in the PBS group. Kidney uptake was significantly lower in the 657 mg/kg group (85.8 ± 24.2 %ID) than in the 329 mg/kg (159 ± 26.2 %ID), 164 mg/kg (211 ± 27.4 %ID), and PBS groups (182 ± 33.5 %ID) (*P* < 0.001). Salivary gland uptake was lower in the 657 mg/kg (3.72 ± 2.12 %ID) and 329 mg/kg (5.74 ± 0.62 %ID) groups than in the PBS group (10.04 ± 2.52 %ID) (*P* < 0.01). **Conclusion:** MSG decreased salivary and kidney uptake of ^68^Ga-PSMA-11 in a dose-dependent manner, whereas tumor uptake was unaffected.

Prostate-specific membrane antigen (PSMA) is an excellent prostate cancer target for theranostic applications. Many imaging agents showing high sensitivity or specificity for PSMA-expressing tissues have been developed (1). Some have been labeled with therapeutic radionuclides (i.e., ^177^Lu and ^225^Ac) and have had success in treating castration-resistant metastatic prostate cancer (2,3). The activity administered to patients is limited by toxicity to normal organs; high uptake is observed in the lacrimal glands, parotid glands, submandibular glands, and renal cortex (4). The potential side effects of higher doses include hematotoxicity, xerostomia, and renal dysfunction (2,3). In particular, xerostomia with α-emitters is so severe that patients have discontinued treatment (5). A means of decreasing this toxicity without affecting tumor uptake would allow administration of greater activity with presumably greater tumoricidal effect.

Different pharmaceuticals, including 2-(phosphonomethyl)pentanedioic acid (PMPA), a PSMA inhibitor, have been explored for nephroprotection (6,7). PMPA displaced renal activity of a PSMA radiotherapeutic in cancer models, but this was generally accompanied by a reduction in tumor uptake (6,7). Mannitol infusion reduced renal uptake of ^68^Ga-PSMA-11 (8), but its effect on tumor uptake requires further investigations. Botulinum toxin was administered to the parotid gland of a patient and significantly decreased PSMA-ligand uptake (9). Although this procedure is promising, it is invasive and costly and may affect salivary gland function for weeks. In this study, we investigated monosodium glutamate (MSG) for reducing uptake of ^68^Ga-PSMA-11 in the salivary glands and kidneys in LNCaP tumor–bearing mice. MSG is a well-studied food additive and can stimulate salivary flow (10,11). Although PSMA is expressed in the salivary glands and kidneys, part of PSMA-ligand uptake in salivary glands may be due to off-target binding, as uptake is not observed in human studies with the radiolabeled J591 monoclonal antibody (12–14). Because many PSMA ligands integrate glutamate for binding to PSMA, we hypothesized that MSG could reduce nonspecific accumulation in noncancerous tissues.

## MATERIALS AND METHODS

### ^68^Ga-PSMA-11 Radiolabeling

The standard and radiolabeling precursor were purchased from ABX Advanced Biochemical Compounds. Radiolabeling was performed as previously published (15).

### Cell Culture

LNCaP prostate adenocarcinoma cells (ATCC) were cultured in RPMI-1640 medium supplemented with 10% fetal bovine serum (Sigma-Aldrich), penicillin (100 IU/mL), and streptomycin (100 μg/m) (Life Technologies) in a humidified incubator (37°C, 5% CO_2_).

### Competition Binding Assay

LNCaP cells were washed with phosphate-buffered saline (PBS) and harvested by trypsinization. Cells (200,000/well) were seeded onto a 24-well poly-d-lysine–coated plate for 48 h. Growth medium was replaced with PBS buffer (with 20 mM 4-(2-hydroxyethyl)piperazine-1-ethanesulfonic acid, 2% bovine serum albumin, pH 7.5) 1 h before the study. ^18^F-DCFPyL (0.1 nM), synthesized according to published procedures (16), was added to wells containing MSG (1 × 10^−2^ to 1.3 × 10^−7^ M), in triplicate. The assays were incubated for 1 h at 37°C with agitation. After aspiration, cells were washed twice with cold 4-(2-hydroxyethyl)piperazine-1-ethanesulfonic acid-buffered saline. To harvest cells, 400 μL of trypsin were added to each well. Samples were counted using a Wizard2 2480 (PerkinElmer) automatic γ-counter. Binding affinity (K_i_) was calculated using nonlinear regression in Prism 7 (GraphPad Software) with a dissociation constant of 0.49 nM for ^18^F-DCFPyL.

### PET Imaging and Biodistribution

Animal experiments were approved by the Animal Ethics Committee of the University of British Columbia. Immunodeficient NOD.Cg-*Prkdc*^*scid*^*Il2rg*^*tm1Wjl*^/SzJ male mice were obtained in-house. Mice were subcutaneously inoculated with 5 × 10^6^ LNCaP cells (100 μL; 1:1 PBS/Matrigel [Corning]), with tumors grown for 4–6 wk. Mice were injected intraperitoneally with MSG (657, 329, or 164 mg/kg) or PBS. After 15 min, mice were anesthetized with isoflurane (2.5% in a 2.0 L/min flow of O_2_) and injected intravenously with ^68^Ga-PSMA-11 (5.34 ± 0.95 MBq for PET/CT, 1.86 ± 0.71 MBq for biodistribution studies).

PET/CT imaging was conducted on a Siemens Inveon small-animal PET/CT device. After a 45-min uptake period, the mice were sedated with isoflurane and placed on the scanner. Body temperature was maintained by a heating pad. A CT scan was obtained for localization and attenuation correction (80-kV tube voltage, 500-μA current, 3 bed positions, 34% overlap, and 220° of continuous rotation). This scan was followed by a 10-min PET acquisition 1 h after injection of ^68^Ga-PSMA-11. PET imaging data were acquired in list mode, reconstructed using 3-dimensional ordered-subsets expectation maximization (2 iterations) followed by a fast maximum a priori algorithm (18 iterations) with CT-based attenuation correction. Images were analyzed using Inveon Research Workplace software (Siemens Healthineers).

For biodistribution studies, 1 h after radiopharmaceutical injection, the mice were anesthetized with isoflurane and euthanized by CO_2_ inhalation. Blood was collected via cardiac puncture. Organs and tissues were harvested, rinsed with PBS, blotted dry, and weighed. The radioactivity in tissues was assayed by γ-counter and expressed as percentage injected dose per gram of tissue (%ID/g).

### Statistical Analysis

Analysis was performed with R, version 3.4.0 (R Foundation for Statistical Computing). Outliers identified by one round of the Grubb test (α < 0.01) were removed. When data distribution was normal according to the Shapiro–Wilk test (α < 0.05), groups were compared using the Welch *t* test or the Wilcoxon rank sum test. The Holm correction was used for multiple comparisons. The significance level was an α value of less than 0.05. PBS controls repeated for each experiment (different batches of radiotracer) were aggregated in a single group for analysis.

## RESULTS

^68^Ga-PSMA-11 was synthesized in 66.3% ± 8.8% decay-corrected radiochemical yields with more than 99% radiochemical purity and 82.6 ± 44.3 GBq/μmol molar activity (*n* = 6).

**FIGURE 1.**
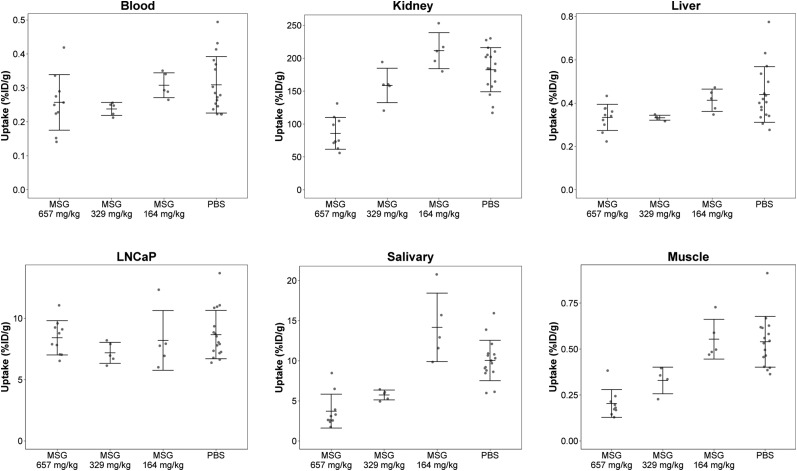
Biodistribution of ^68^Ga-PSMA-11 in selected organs at 1 h after injection. Mice received MSG (657, 329, or 164 mg/kg) or PBS intraperitoneally 15 min before tracer administration.

Biodistribution results are shown in Figure 1 and Supplemental Table 1 (supplemental materials are available at http://jnm.snmjournals.org). There were no statistical differences for tumor uptake between the 4 groups, at 8.42 ± 1.40 %ID/g in the 657 mg/kg group, 7.19 ± 0.86 %ID/g in the 329 mg/kg group, 8.20 ± 2.44 %ID/g in the 164 mg/kg group, and 8.67 ± 1.97 %ID/g in the PBS group. Kidney uptake was significantly lower in the 657 mg/kg group (85.8 ± 24.2 %ID/g) than in the groups given 329 mg/kg (159 ± 26.2 %ID/g), 164 mg/kg (211 ± 27.4 %ID/g), and PBS (182 ± 33.5 %ID/g) (*P* < 0.001). The difference between the 329 and 164 mg/kg groups was significant (*P* < 0.05), but not when they were, respectively, compared with PBS. Salivary gland uptake was lower in the 657 mg/kg (3.72 ± 2.12 %ID/g) and 329 mg/kg (5.74 ± 0.62 %ID/g) groups than in the PBS group (10.04 ± 2.52 %ID/g); *P* < 0.01. There were no significant differences between the 164 mg/kg (14.2 ± 4.27 %ID/g) and PBS groups, or between the 657 and 329 mg/kg groups. Muscle uptake was generally lower in MSG-treated groups.

**FIGURE 2.**
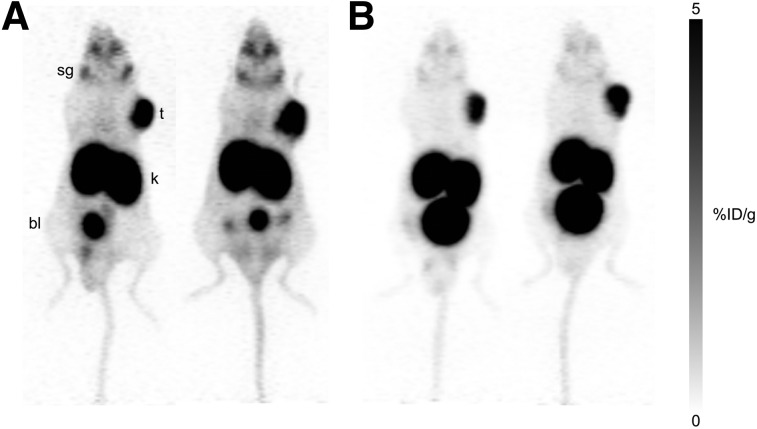
PET maximum-intensity projections of 4 mice with ^68^Ga-PSMA-11 at 1 h after injection. LNCaP tumor–bearing mice received PBS (A) or MSG (B) (657 mg/kg) intraperitoneally 15 min before tracer administration. bl = bladder; k = kidney; sg = salivary glands; t = tumor.

PET imaging studies were performed on mice preinjected with PBS or a 657 mg/kg dose of MSG (Fig. 2). High uptake in LNCaP tumors was observed, with the remainder of the radioactivity cleared through the renal pathway. Compared with the PBS group, MSG-treated mice showed lower uptake in the salivary glands and faster excretion (more urinary output). The difference in clearance rates led to higher-contrast images for MSG-treated mice.

**FIGURE 3.**
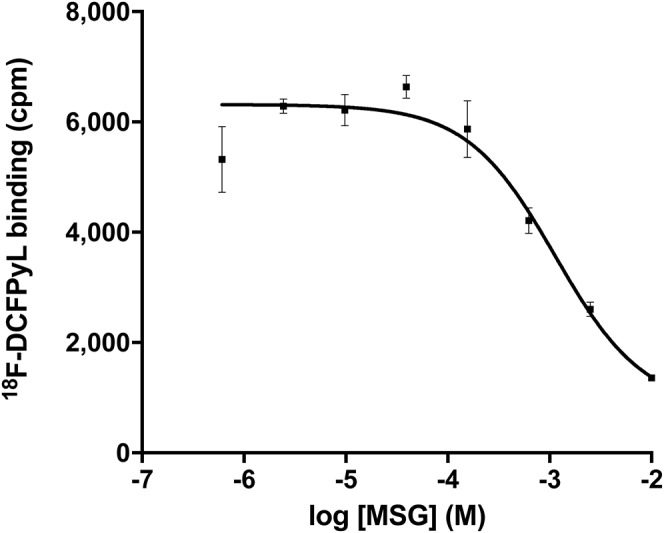
Representative competition binding assay for MSG against ^18^F-DCFPyL in LNCaP cells. K_i_ value for this assay was 0.95 mM.

The binding affinity (K_i_ value) of MSG to PSMA was 0.90 ± 0.58 mM (*n* = 3), as measured using a cell-based competition assay with ^18^F-DCFPyL (Fig. 3).

## DISCUSSION

The development of PSMA radiotheranostic agents has had a significant impact on prostate cancer management (17). Small-molecule inhibitors were developed as alternatives to monoclonal antibodies, primarily for their fast targeting properties and clearance profiles (17). However, radiolabeled PSMA inhibitors show undesired uptake in salivary glands (17). Although this does not hinder diagnostic interpretation, it imposes a limit on the maximum-tolerable dose for therapy.

We investigated the effect of MSG on ^68^Ga-PSMA-11 salivary uptake. The doses of MSG chosen for this study corresponded to one tenth, twentieth, and fortieth of the median lethal dose for mice (18).

We observed a significant decrease in kidney uptake (50% for 657 mg/kg) and salivary uptake (43%–53% for 329 and 657 mg/kg) of ^68^Ga-PSMA-11 for MSG-treated mice compared with control. Uptake in kidney and salivary glands was higher for the 164 mg/kg group than for the PBS group, but this difference was not statistically significant. Notably, tumor uptake did not significantly differ among the 4 groups. At more than 329 mg/kg, MSG blocked renal and salivary uptake without affecting tumor uptake. PET imaging corroborated the biodistribution results, notwithstanding enhanced contrast for MSG-treated mice, which can be explained by accelerated clearance of ^68^Ga-PSMA-11 or reduced nonspecific accumulation.

Kratochwil et al. demonstrated that PMPA can reduce renal uptake of ^125^I-MIP-1095 without affecting LNCaP tumor uptake; however, a 16-h latency period was required for tracer binding and clearance before PMPA administration (6). Chatalic et al. showed that coinjection of PMPA with ^111^In/^177^Lu-PSMA I&T could improve the tumor-to-kidney absorbed dose ratio, but this improvement was accompanied by a reduction in tumor uptake (7). The high affinity of PMPA to PSMA (reported K_i_, 0.28 nM) may complicate dose optimization (19). Conversely, MSG has poor binding affinity to PSMA (K_i_, 0.90 ± 0.58 mM), which may benefit this application.

Although MSG has been linked to headaches and “Chinese restaurant syndrome,” human studies have shown MSG to be safe; based on a no-adverse-event limit of 3,200 mg/kg/d for neurodevelopmental toxicity, the European Food Safety Authority advised that a daily intake of 30 mg/kg is acceptable (18,20). Fernstrom administered a single 12.7-g dose of MSG orally to humans without side effects, with a return of plasma glutamate levels to baseline after 3 h (21). The MSG quantity required to achieve effective off-target blocking of ^68^Ga-PSMA-11 in humans is not known, as human physiology may vary. Whether sufficient quantities can be practically administered to human subjects will require further investigation.

## CONCLUSION

MSG can decrease salivary and renal uptake of ^68^Ga-PSMA-11 without affecting tumor uptake in mice, presumably by competing with off-target binding sites. This effect could potentially be used to increase the therapeutic index of glutamate-based radioligands. Further work is needed to assess whether this blocking effect is sufficiently durable to protect these organs from longer-lived radioisotopes, to understand the mechanism of action and to assess if the same effect can be translated to humans.

## DISCLOSURE

Financial support was provided by the Canadian Institutes of Health Research (FDN-148465), the BC Cancer Foundation, and the BC Leading Edge Endowment Fund. No other potential conflict of interest relevant to this article was reported.
