# Supplementary material for: Monosodium Glutamate Reduces 68Ga-PSMA-11 Uptake in Salivary Glands and Kidneys in a Preclinical Prostate Cancer Model
Source: J Nucl Med. 2018 Dec;59(12):1865–8. doi: 10.2967/jnumed.118.215350 (PMC6278899; doi:10.2967/jnumed.118.215350)
Supplement: Supplementary file 1 [file jnm215350SupplementalData.pdf]

Supplemental Table 1. Effect of MSG on biodistribution of ^{68}Ga-PSMA-11 in LNCaP tumor-bearing mice at 1 h post-injection.

	MSG 657 mg/kg		MSG 329 mg/kg		MSG 164 mg/kg		PBS	
	**mean±SD**	**n**	**mean±SD**	**n**	**mean±SD**	**n**	**mean±SD**	**n**
**Blood**	0.26±0.08	10	0.24±0.02	5	0.31±0.04	5	0.31±0.08	17
**Fat**	0.65±0.20	10	1.14±0.25	5	2.49±0.96	5	2.25±0.75	17
**Spleen**	4.31±1.98	10	7.91±3.44	5	19.5±6.19	5	22.2±7.91	17
**Liver**	0.33±0.06	10	0.33±0.01	5	0.41±0.05	5	0.44±0.13	17
**Pancreas**	0.33±0.05	9	0.57±0.11	4	1.21±0.33	5	1.44±0.39	17
**Adrenals**	2.45±1.20	9	5.30±3.61	3	8.67±2.16	5	7.47±2.36	13
**Kidneys**	85.8±24.2	10	159±26.2	5	211±27.4	5	182±33.5	17
**Muscle**	0.20±0.08	9	0.33±0.07	4	0.55±0.11	5	0.54±0.14	16
**Tumor**	8.42±1.40	10	7.19±0.86	5	8.20±2.44	5	8.67±1.97	17
**Salivary glands**	3.72±2.12	10	5.74±0.62	5	14.2±4.27	5	10.0±2.52	16
